# Stereo-Encephalographic Presurgical Evaluation of Temporal Lobe Epilepsy: An Evolving Science

**DOI:** 10.3389/fneur.2022.867458

**Published:** 2022-05-27

**Authors:** Elma Paredes-Aragon, Norah A. AlKhaldi, Daniel Ballesteros-Herrera, Seyed M. Mirsattari

**Affiliations:** ^1^Department of Clinical Neurological Sciences, Western University, London, ON, Canada; ^2^Neurology Department, King Fahad Hospital of the University, Imam Abdulrahman Bin Faisal University, Dammam, Saudi Arabia; ^3^Neurosurgery Department, National Institute of Neurology and Neurosurgery “Dr. Manuel Velasco Suárez”, Mexico City, Mexico; ^4^Departments of Clinical Neurological Sciences, Diagnostic Imaging, Biomedical Imaging and Psychology, Schulich School of Medicine and Dentistry, Western University, London, ON, Canada

**Keywords:** intracranial EEG, epilepsy, seizure, epileptogenic zone, epilepsy surgery

## Abstract

Drug-resistant epilepsy is present in nearly 30% of patients. Resection of the epileptogenic zone has been found to be the most effective in achieving seizure freedom. The study of temporal lobe epilepsy for surgical treatment is extensive and complex. It involves a multidisciplinary team in decision-making with initial non-invasive studies (Phase I), providing 70% of the required information to elaborate a hypothesis and treatment plans. Select cases present more complexity involving bilateral clinical or electrographic manifestations, have contradicting information, or may involve deeper structures as a part of the epileptogenic zone. These cases are discussed by a multidisciplinary team of experts with a hypothesis for invasive methods of study. Subdural electrodes were once the mainstay of invasive presurgical evaluation and in later years most Comprehensive Epilepsy Centers have shifted to intracranial recordings. The intracranial recording follows original concepts since its development by Bancaud and Talairach, but great advances have been made in the field. Stereo-electroencephalography is a growing field of study, treatment, and establishment of seizure pattern complexities. In this comprehensive review, we explore the indications, usefulness, discoveries in interictal and ictal findings, pitfalls, and advances in the science of presurgical stereo-encephalography for temporal lobe epilepsy.

## Introduction

Temporal lobe epilepsy (TLE) is responsible for 30% of cases of drug-resistant epilepsy (DRE), the largest subgroup of DRE cases. Location of the epileptogenic zone (EZ) associated with a lesion, such as mesial temporal sclerosis (MTS), is a high predictor of good seizure outcome (1). Usual arrival to a final hypothesis of the seizure onset zone involves the work of a multidisciplinary team of experts (neuropsychologist, neurosurgeon, psychiatrist, epileptologist, and neuroradiologist), favoring less invasive procedures for the determination of a surgical plan (2). Determination of the EZ is complex and with less invasive scalp electroencephalogram (EEG), discordant data may complicate the surgical treatment of DRE (3). Studies in the past have shown that when correlating surface EEG findings with simultaneous subdural and intracranial findings of brain structures, most subclinical seizures originating from the hippocampus did not result in surface/scalp EEG changes (4). Approximately 25% of patients with DRE have no conclusive non-invasive scalp EEG, imaging, and investigations in the process of identifying the EZ (2). Thus, the need for more invasive procedures as a part of the study of TLE in DRE has substantially complemented non-invasive workup.

Invasive procedures, such as subdural electroencephalography (SDEEG), were once used in Comprehensive Epilepsy Centers; as technology advanced, tendencies have shifted to intracranial recordings using stereo-encephalography (SEEG) in most centers. In this comprehensive review, we discuss classic and novel approaches for the study of TLE and advances in presurgical planning using SEEG, as well as controversial aspects in this growing field.

## History OF Stereo-Encephalography in the study of Temporal Lobe Epilepsy

Distinct from superficial cerebral cortical analysis applied by Wilder Penfield at the Montreal Neurological Institute, the SEEG analysis of brain structures began initially with Spiegel and Wycis (5). Their approach of stereotaxis began in 1947 and was applied in Paris for the investigation of TLE. The culmination of the insertion of depth electrodes for the study of deep brain structures was reached by Bancaud and Talairach in 1957 and then perfected 10 years later (6). The possibility of three-dimensional analysis of brain structures with a stereotactic atlas has yielded marvelous advances in the study of deep brain involvement in epilepsies.

The mechanisms of TLE were also enriched by SEEG. Starting with Jasper's initial description of EEG suppression preceding seizures originating from mesial foci, the interhemispheric connections of both temporal regions associated with temporal region pathology pushed for invasive studies. With the help of Bancaud and Talairach's Atlas, correlation of location with clinical manifestations, onset, and propagation of brain activity was possible. This made it tangible to study the seizure pattern both ictally and interictally (7). Challenging Jasper's “irritative zone” concept as the origin of seizures, they considered this zone interictal. Instead, they found that the seizure onset zone (SOZ) could in fact be far away from the irritative zone. As we now know, this concept changed the study of epilepsy, and the notion of seizures as an epileptogenic network was born (8). Finally, Crandall described specific changes in SEEG associated with hippocampal sclerosis and then conducted microelectrode recordings for microcircuits, and unit activity and study epileptogenic circuits in the mesial temporal regions (9).

## SEEG in the Study of Temporal Lobe Epilepsy Networks

As Bancaud and Talairach depicted, epilepsy networks in TLE are not solely located in the temporal lobe, but rather involve a widespread region of structures. This important description is key to understanding seizure phenomena. SEEG has contributed to our knowledge of TLE circuits. In a study involving 18 patients with DRE and TLE that underwent SEEG, Bartolomei et al. used a non-linear correlation method to measure the degree and direction of coupling in the SEEG signal. Importantly, this study included MTLE only, excluding lateral epilepsy cases and possible temporal-plus patients. They analyzed the functional coupling between 3 regions of the TL: the anterior temporal neocortex, the amygdala, and the anterior hippocampus. In 10 patients, the ictal discharges were limited to only mesial limbic structures with propagation secondarily to the cortex. In contrast, there was a constant coupling between the hippocampus and amygdala. In 5 patients, medial-lateral networks were identified, and in 3 patients lateral-medial networks were identified; both groups had initial ictal discharge in limbic and neocortical regions, with a rapid “tonic” discharge (10).

In a recent study with SEEG and MRI diffusion tensor imaging (DTI), 33 patients with TLE were included. The objective of the study was to establish the directionality of the seizure spread and the involvement of white matter tracts. Directionalities were divided into anterior-posterior or medial-lateral based on SEEG interpretations and DTI connectome and fractional anisotropy. Medial-lateral spread was found to have more fractional anisotropy in the corpus callosum and to lesser degrees both cingulate tracts; in contrast, antero-posterior seizures had fractional anisotropy along the cingulate fasciculus and inferior longitudinal fasciculus, with scarce involvement of the corpus callosum. Thus, the group showed that white matter tracts are key in epileptogenic TLE networks (11). These findings may yield future treatment of TLE with possible disruption of the network for patients not amenable to surgery.

## Indications

**Figure 1:**
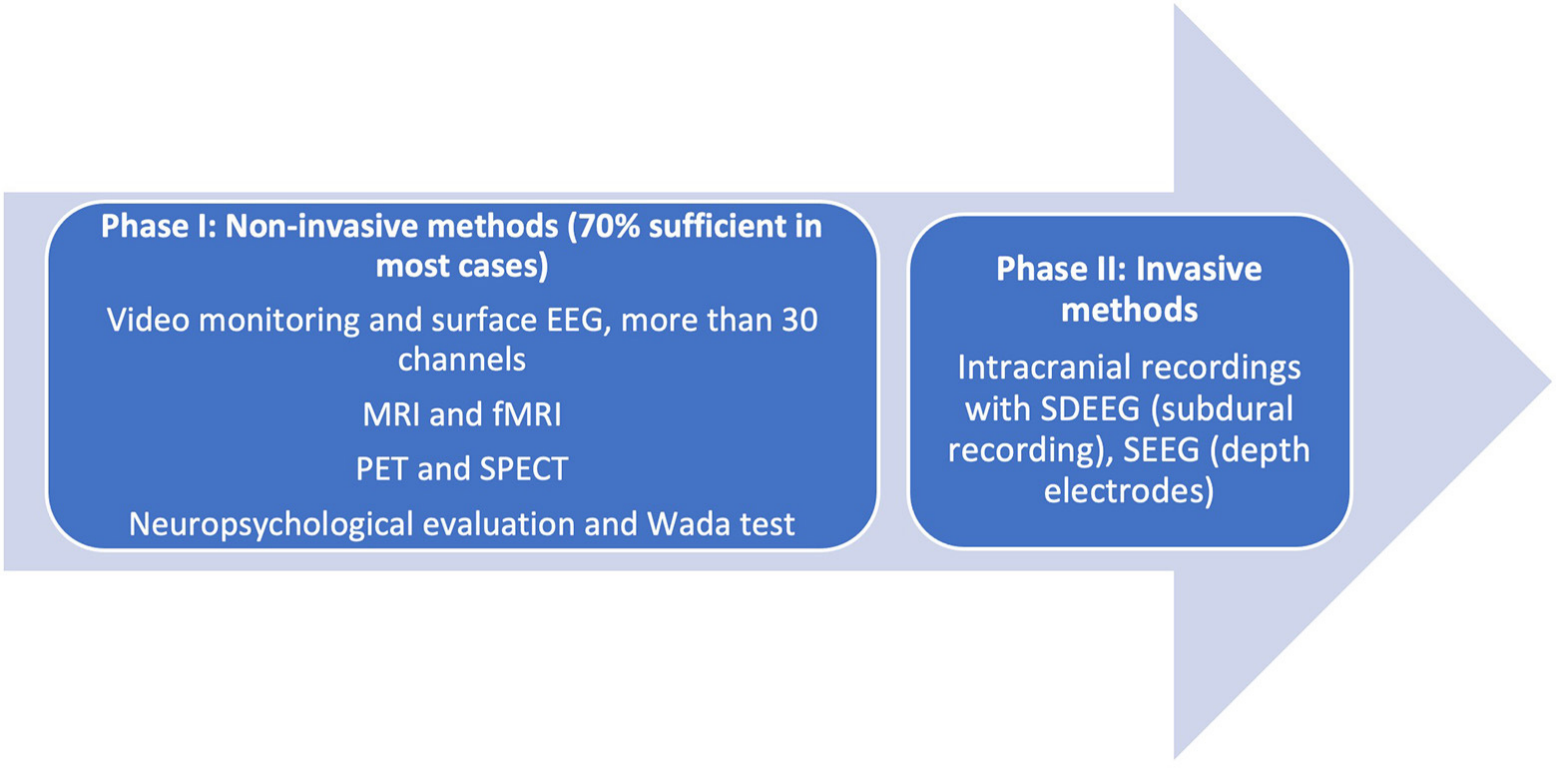
Phases of presurgical evaluation of epilepsy. [Adapted from (12)]. EEG, electroencephalography; MRI, magnetic resonance imaging; fMRI, functional magnetic resonance imaging; SDEEG, Subdural electroencephalography; SEEG, Stereoencephalography.

Prior to consideration of SEEG recording, it is imperative to remember that presurgical investigations have two main objectives: (a) localization of the epileptogenic process [composed of the epileptogenic lesion, interictal activity in the EEG (irritative zone), ictal zone (epileptogenic zone)] and (b) preparation for surgical planning boundaries. In some cases, these are met in the first phase of investigation (Phase I, Non-Invasive), when clinical, electrical, and imaging information is concurrent with a hypothesis for localization. In these cases, the algorithm ends here. Phase II of studies is reserved for cases where data are conflicting in semiology, EEG, and additional Phase I methods of investigation. The unknown precise location within a hemisphere or difficulties in lateralization, visible lesions in MRI, electroclinical data, or functional imaging may warrant invasive investigations (Figure 1).

Subdural electroencephalography provides a two-dimensional image of the brain structures and SEEG provides a three-dimensional view. While SDEEG is followed by a determined surgical decision, SEEG provides different information on the following: resection of the EZ and complexity of the eloquent cortex (EC), ablation of a single contact involving the EZ, withholding a resection if risks are elevated (5). In general, patients that do not undergo resection after SEEG implantation and recording is up to 40% (2, 13). Finally, in patients with non-lesional MRIs that have multifocal seizure origin during Phase I studies, invasive monitoring should be generally avoided (4).

Indications of intracranial recording of cerebral activity or SEEG have shifted throughout the years. The optimal suggested method of decision-making for invasive recording using SEEG is through a multidisciplinary process that involves insight from all members of the group: psychologists, neuropsychologists, epileptologists, epilepsy neurosurgeons, neuroradiologists, nurses, and social workers.

In general, invasive recordings are warranted to 1. define the EZ precisely when non-invasive data are inconclusive (rapidly generalizing seizures, differentiating between lobar or regional epilepsy, determining if a seizure is a temporal or temporal plus, determining if onset is mesial versus neocortical, dual pathology, or determining if there is a dysplasia); 2. resolve diverging information in non-invasive data that directs in two different regions (bilateral mesial temporal foci, large lesions, encephalomalacia, multiple lesions, such as tuberous sclerosis, and nodular heterotopia) 3. Map eloquent functional cortex, and 4. To further corroborate the EZ, to gain prognostic information, and ablate regions using thermocoagulation (14).

Specific indications most frequently applied in the field of TLE include (2, 13, 15):

- Differentiating mesial from neocortical involvement in EZ
- Extension of the EZ beyond the temporal region
- The bitemporal onset of epilepsy requires exploring mesial temporal structures that may or may not include the hippocampus (although most centers cover this area) for bilateral intracranial recording to determine the laterality of seizures.

**Figure 2:**
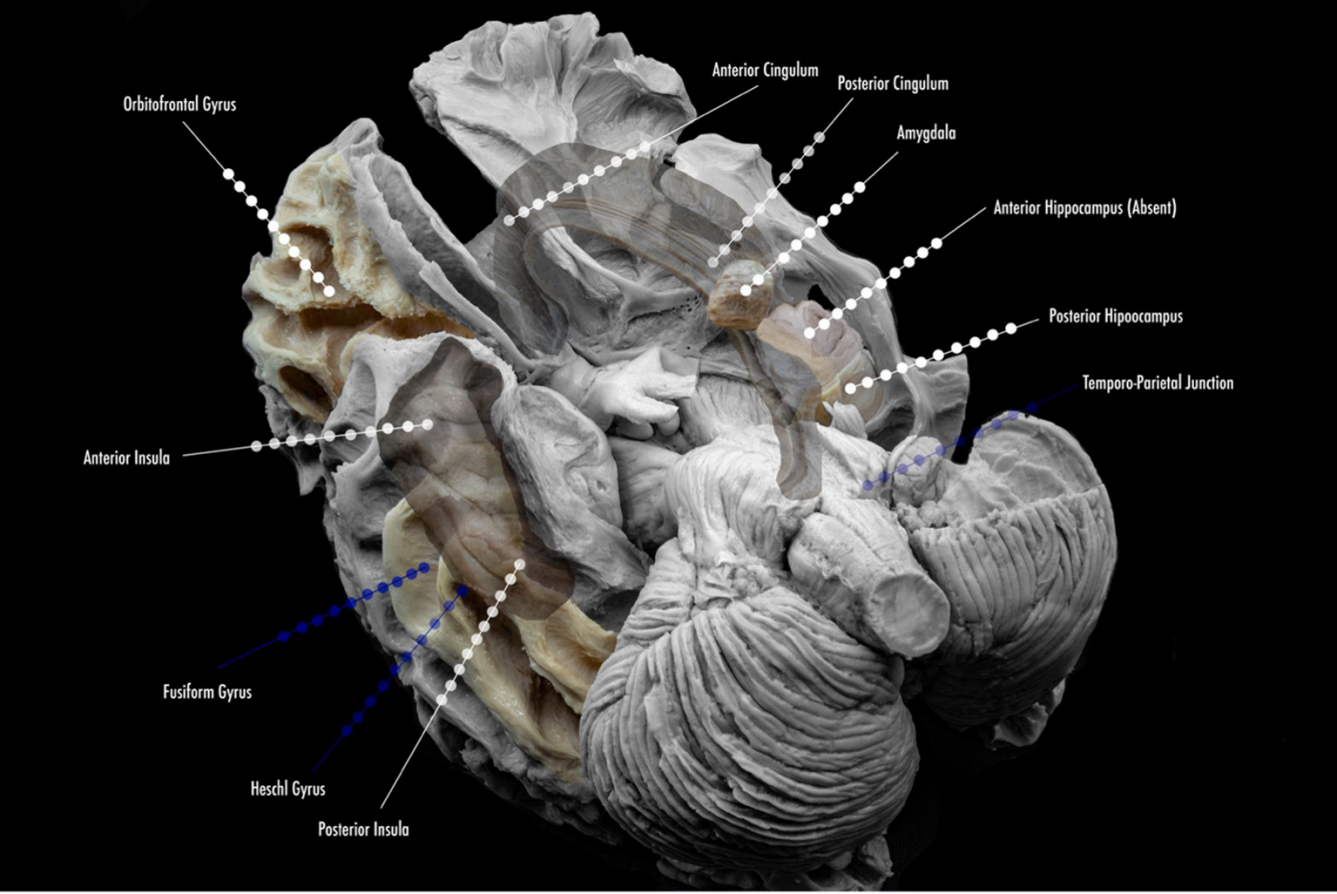
Proposed scheme of electrode implantation maps for insertion. Lined white dots show usual trajectories and sites of insertion in the study of temporal lobe epilepsy. Lined blue dots show additional electrode locations for the study of temporal plus epilepsies, and common sites of propagation.

More specific details about indications of SEEG in TLE will be addressed in the following subsections of this article. The suggested mapping schematic when the hypothesis is TLE is illustrated in Figure 2.

## SDEEG vs. SEEG in the Study of Temporal Lobe Epilepsy

While much of the available literature favors SEEG over SDEEG, discordant information exists in the study of TLE. This lies in the fact that the neocortical TLE is adequately viewed with SDEEG. The pattern of spread through the neocortex is also adequately and broadly appreciated with SDEEG. However, SEEG is superior in the cases of mesial involvement, or when suspicion of temporal-plus epilepsy is involved, likely requiring insular coverage. Indeed, SEEG is far superior for cases where sulci are involved more than gyri (16). Surgical outcomes have rendered SEEG non-inferior to SDEEG. In seizure freedom outcomes, SEEG is superior to SDEEG (61 vs. 56.4%) (17). In general, SEEG is well-tolerated. It also conveys lower morbidity (4.8%) and mortality (0.2%) when compared to SDEEG (15.5 and 0.4%, respectively) (18).

In general, the majority of North American Comprehensive Epilepsy Centers have moved toward SEEG in all phase II investigations. In select cases, some centers may prefer to explore epilepsy caused by superficial cortical lesions with SDEEG. New information has yielded importance in SEEG with lesional epilepsy; it will be addressed in a separate section. SEEG also permits obtaining details about areas, such as multiple lobes, or remote locations that are surrounding the EZ for planning without the need for craniotomies (2, 5, 17–20). Finally, extra-operative cortical stimulation (CS) for mapping in SDEEG has the advantage of allowing coverage and sampling of the superficial cortex, but SEEG in the exploration of the TLE and the limbic system holds true to a more precise recording and mapping of additional limbic structures: insular cortex, orbitofrontal cortex, and the cingulate gyrus (Figure 2) (2, 5, 19–21).

## Technical Details of SEEG: Sampling Rate, Acquisition of Data, Center Recommendations, and Other Distinctions

Before the insertion of depth electrodes for SEEG recording, recommendations were published and standardized for the centers that were recommended to apply these techniques. The success of surgical treatment and implantation depends especially on the experience and number of patients an epilepsy center reaches per year. An estimated (and gold standard according to some groups) is 20 patients per year or 50 surgeries in 4 years (5, 14). Some European centers have established a minimum of 5 SEEG procedures per year to be regarded as a center of excellence (22). The ILAE Commission on Neurosurgery of Epilepsy suggests establishing the term “basic centers” for hospitals that have limited resources and “reference centers” for hospitals that have the experience of performing established presurgical and surgical management (5, 20, 23).

After careful SEEG planning by a multidisciplinary team, (specified elsewhere in this review) targets are reached using depth electrodes that are commercially available according to the country/region. These are implanted using the standard stereotactic technique, and in some centers with the assistance of a stereotactic robot, drilling 2.5 mm diameter holes. Using orthogonal or oblique orientations depending on the center permits dynamic recording of cortical, subcortical, lateral, intermediate, or deep structures. All these elements permit an adequate 3-dimensional view of the epileptogenic zone and the surrounding structures of interest, and upon recording, the spatiotemporal interaction of these regions when seizures occur.

**Figure 3:**
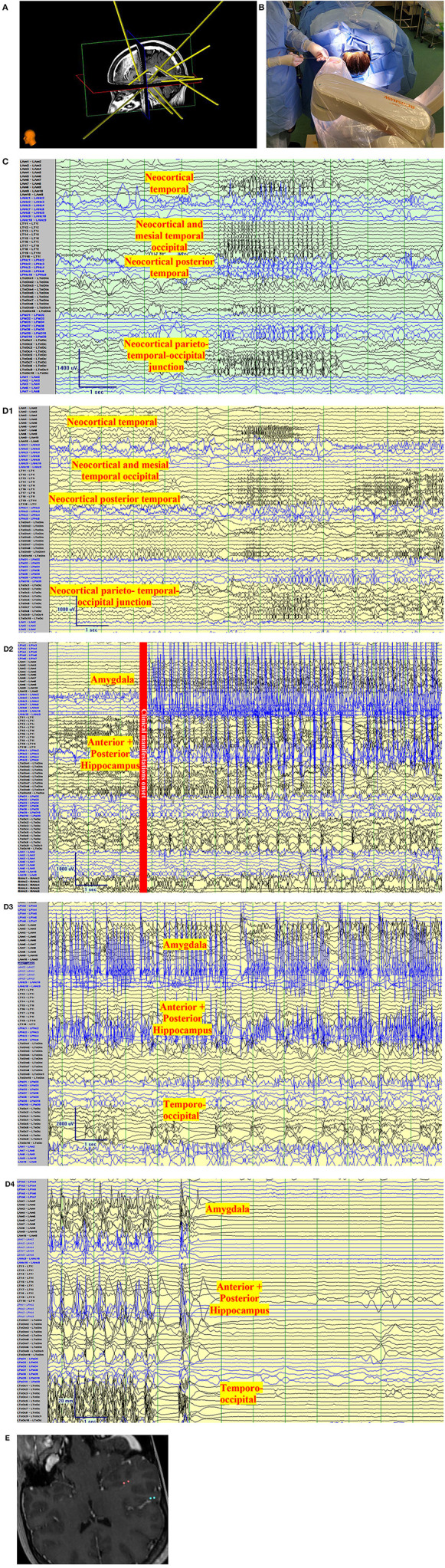
Clinical vignette. A 24-year-old left-handed female, like her father, began with epilepsy at age 15. Semiology began as a non-specific abdominal sensation, loss of awareness, speaking “gibberish”, or changing subjects of conversations. The seizures were often associated with lacrimation, salivation, and chewing automatisms with a frequency of 2 seizures per month. There was no tendency to progress to bilateral tonic-clonic. She has tried more than 7 antiseizure medications. Interictal Scalp encephalography (EEG) findings in Epilepsy Monitoring Unit admissions were unremarkable with no interictal discharges with the exception of rare left posterior temporal polyspikes during sleep (T5 spreading to O1-T3). Ictal Scalp EEG showed onset over the same area, but the scalp EEG changes did not precede clinical manifestations by more than 30 s. MRI Brain, was unremarkable, positron emission tomography (PET) CT showed no definite quantitative or qualitative hypometabolic focus. SPECT showed no clear focal area of hyperperfusion but showed a non-specific increase in perfusion in the left temporoparietal region. The neuropsychological evaluation showed left-hemispheric language dominance and mild memory impairment. The case was presented to a multidisciplinary team and stereo-encephalography (SEEG) implantation was decided. Limbic coverage with an emphasis on the temporo-parietal occipital junction (due to the EEG, SPECT, neuropsychological findings) and the opercular-insular region (because of clinical findings) was decided. After presurgical investigations were completed, resective surgery was not decided due to the risk of global aphasia. **(A)** Trajectory planning of SEEG electrode insertion with Renishaw Neuroinspire™ software, co-registered with MRI. *(Image courtesy of Dr. David Steven and Dr. Greydon Gilmore, London Health Sciences Centre, London, Ontario, Canada)*. **(B)** Insertion of Depth electrodes by Renishaw Neuro-Mate. Neurosurgeon and robot assisting device when installing depth electrodes in the operating room. *(Image courtesy of Dr. David Steven and Dr. Greydon Gilmore, London Health Sciences Centre, London, Ontario, Canada)*. **(C)** Interictal SEEG: Longitudinal bipolar montage of intracranial SEEG recording with a sampling rate of 1,280, showing synchronized spikes seen as runs lasting up to 4 s at a time involving the neocortical temporal, temporo-occipital, and parietal-occipital region. Synchronic interictal findings are a frequent finding of temporal lobe epilepsy and its connections. **(D)** Ictal SEEG: **(D1)** Longitudinal bipolar montage of intracranial SEEG recording with a sampling rate of 1,280, showing an attenuation of the background activity, with low voltage fast activity in the same regions of synchronization seen in A for 5-6 s. **(D2)** Previously seen attenuation is followed by a high-voltage spike that runs over the mesial temporal regions. **(D3)** As the seizure propagates, the activity spreads to the neocortical temporal regions. **(D4)** Periods of attenuation occur until the final offset is seen as attenuation. **(E)** Cortical stimulation showed a wide hyperexcitable epileptogenic network involving the parieto-temporo-occipital region, both mesial and neocortical. When stimulating the left mesial temporal region, anomia occurred (pink color in co-registered MRI) and when stimulating the neocortical temporo-occipital region, speech arrest occurred.

On the day of the surgery, the patient is admitted with a previously established plan of frame-based implantation with a volumetric T1 sequence contrast-enhanced MRI (15, 21, 24). These images are transferred to a neuro-navigation software (varies according to center, in our center we use Renishaw Neuroinspire™ Software, Missisauga, Ontario, Canada, https://www.renishaw.com/en/neuromate-robotic-system-for-stereotactic-neurosurgery–10712; https://www.renishaw.com/en/neuroinspire-neurosurgical-planning-software–8244), (Figure 3) planning trajectories and targets of insertion and matching with vessel trajectories to prevent bleeding complications (18). Under general anesthesia, a stereotactic frame is placed and confirmed with a CT angiogram. The implantation in most large Comprehensive Epilepsy Centers has shifted toward the use of a robotic implantation device, Renishaw™ in our center (Figure 3). This robot has a cannula that measures 2.5 mm in diameter and is secured to the device arm. Dura is perforated and 2 mm diameter holes are done, with an insertion of an implantation bolt. A small stylet (2 mm in diameter) is inserted into the burr hole site and passed gently into the brain, guided by the initial implantation bolt (2). The depth electrodes used in SEEG recording are strands of cylindrical contacts (ranging from 4 to 18), spaced 2–10 mm apart with a diameter of 1 mm or less and recording areas of 3–5 mm^2^. The electrodes can be rigid or semi-rigid depending on the center (14). Robotic assistance has proven more time-efficient and just as precise as conventional stereotactic surgery (24, 25). Control CT or MRI (if electrodes are compatible) is taken to ensure no acute complication occurs. (Complications are explained elsewhere in this article).

The EEG system must be able to support at least 128 channels (ideally 256 channels), 50 or 60 Hz filter is highly discouraged, as it could affect low voltage fast activity (LVFA) and the identification of high-frequency oscillations (HFOs). No standard nomenclature exists to label each electrode, but names referencing the deep area studied are used (for example, RAHc: Right anterior hippocampus). When comparing the electrode readings with the co-registered MRI software, contacts located in white matter or close to the dura should be excluded. Simultaneous and synchronous recording of scalp EEG is not necessary, but it can be performed by means of a few electrodes. The sampling frequency must be at least 256 Hz (ideally ≥512 Hz or ≥1,024 Hz). Most Comprehensive Epilepsy Centers use a minimum sampling rate of 1,280 Hz for the appropriate evaluation of the HFOs (22).

## Implantation Planning

Perhaps the most important portion of the SEEG implantation process is the planning strategy. A hypothesis is generated ideally with a multidisciplinary team considering the clinical seizure lateralizing and localizing signs, as well as the imaging and nuclear medicine findings, neuropsychological language, memory testing results, and psychiatric comorbidities. The concept of three-dimensionality comes into play in this step of the process, as we consider the symptomatogenic zone (lateralizing and localizing signs, early and postictal), the irritative zone (interictal and ictal epileptiform abnormalities), and the eloquent cortex. The implantation of depth electrodes does not simply convey information about the seizure onset and propagation, but also informs the team of the interactions of the EZ with the eloquent cortex, to establish resection limits. Depth electrodes should strive to sample the anatomic lesion of interest (if there is one), ictal onset structures, early and late spread regions, and the interaction with functional regions (26).

When exploring the temporal regions for epilepsy, one should keep in mind that the temporal region interacts and connects with several extratemporal areas. SEEG must at times require coverage of these areas to determine spread to these regions, or in specific cases where neocortical temporal findings are seen. In some cases, with non-invasive findings, extratemporal epilepsy can go undetected as deeper structures are involved, especially the insula (15). There is no standardized planning scheme, as has been suggested thoroughly by many authors worldwide. Most centers in North America cover the temporo-insular-anterior perisylvian areas, and/or temporo-insular-orbitofrontal areas, or posterior temporal-posterior insula, temporobasal, parietal, and posterior cingulate areas (2). The practicality of such implantation is debated. A highly individualized standard of implantation must be discussed, but a standard “Limbic” coverage is necessary in some cases.

Suggested coverage in our center usually involves the anterior and posterior hippocampus, amygdala, and anterior cingulate gyrus. In most cases, coverage is extended to the anterior, middle, and posterior insula, orbitofrontal region, and posterior cingulate gyrus, as part of the limbic system and its connections. In special cases, the exploration might include strong neocortical characteristics (according to non-invasive findings) and might extend to Heschl's gyrus (with semiology that suggests this region), the fusiform gyrus, the temporo-parietal-occipital junction (when language and vision are also affected, or neocortical semiology is present). Figure 3 depicts the anatomical location of frequent sites of implantation within the limbic system, with a focus on TLE. The individual findings are exclusive to every case, and one cannot use standard implantation for all patients.

## SEEG Interpretation: Back to Basic Principles

As the evaluation of SEEG is 3-dimensional, one cannot talk about SEEG interpretation without previously establishing basic principles in the study of epilepsy, in this case, temporal lobe epilepsy (2, 7, 19, 20). SEEG follows basic principles of interictal activity (interaction of lesion in parenchyma, and epileptiform abnormalities) combined with ictal activity. The fundamental zones involved in the epileptogenic network come into play, that within the SEEG “reading system” may coexist: lesional, interictal, and ictal zones (Table 1). The definition of “seizure onset zone” mainly used in SDEEG differs, as it consists only of measuring latencies between contacts, no matter what the seizure pattern or frequency. Another term is used in SEEG, “early spread network” referring to the early or late propagation of activity, that regards the electrical and semiology network of a seizure (28). With this 3- dimensional view, the interpretation of SEEG requires reading and interpreting by an experienced epileptologist or neurophysiologist. As with less invasive procedures during phase I studies, clinical characteristics of seizures follow the same mainstays of localizing and lateralizing signs as part of the symptomatogenic zone, which may be close or farther away from the EZ or LZ.

**Table 1:** Definition of some commonly used terminology in stereo-encephalography (SEEG) study.

**Concept**	**Definition**	**Example**
Lesional Zone (LZ)	Site(s) of permanent slow background activity, independent of seizure recurrence.	Tumor, stroke, gliosis, etc.,
Irritative zone (IZ)	Site(s) of abnormal interictal paroxysmal activities. Waveforms may be various and complex. Rarely focal in TLE but spread within cortico-cortical networks.	Spikes, sharp waves, spike and waves, low voltage fast activity.
Second Irritative Zone	A zone that is spatially distinct from the seizure onset and occurrence dependent on primary spikes.	Frequently seen in Temporal lobe epilepsy as synchronic spikes. (See Figure 1).
Epileptogenic Zone (EZ)	Site(s) of “primary organization” of ictal discharge. Triggering this area with cortical stimulation will in theory cause a seizure.	
High Frequency Oscillation (HFO)	High frequency interictal and ictal activity in gamma range that has high correlation with the epileptogenic tissue and zone (27)	Seen frequently in focal cortical dysplasia and lesional epilepsy
Afterdischarges (AD)	A seizure pattern following single or repetitive electrical stimulations of a discrete area of the brain by using intracerebral electrodes.	No specific pattern seen; EEG finding is similar to IZ.

## Interictal SEEG in Temporal Lobe Epilepsy

When reading a recording, important details must be noticed, such as the recording rate at which the SEEG is done (see section above), and observation of the location of each contact. Evaluation of the location of every contact previous to reading and ultimately interpreting SEEG, as this represents a 3-dimensional view. Basic principles, such as abundance, still apply, but temporospatial distribution and the relationship between electrodes in seizures also must be studied. For instance, the presence of interictal discharges may be present in the mesial temporal region, but also simultaneously in the anterior insula. This represents a synchronous activation of different structures in the limbic system. This finding is not rare in limbic epilepsy. In addition, an abundance of interictal spikes or discharges is relevant, as well as well-described paroxysmal fast activity that can indicate a lesional finding (2).

Interictal activity for the limbic system has been described and must be distinguished from abnormal background activity. With the development of the brain atlas of normal SEEG recordings in Montreal using 106 subjects, characteristics of specific brain regions were established using SEEG and quantitative recordings. Interestingly, alpha activity was seen in a lower frequency in the temporal lobe (7.75–8.25 Hz) when compared to the occipital region. In addition to well-known information regarding theta findings, the temporal lobe had delta activity as well, (0.75–2.25 Hz) maximum over the hippocampal region (16).

No specific pattern for temporal lobe epilepsy interictal activity has been described in the literature. Some authors consider postictal and interictal activity alone insufficient to detect the lesional and “irritative” zones (29). Contradicting these findings, a group in France attempted to characterize the pathologic process of interictal activity as a potential biomarker. They found that epileptic spikes were in close relation to the EZ. Interestingly, they can also appear in regions remote from the EZ. This “secondary irritative zone” manifests the propagation of the epileptogenic network (30). A study using SEEG found that the distribution of interictal epileptic spikes using the spike frequency index and the topography of the EZ at the time differed in neocortical epilepsies (31). The concepts of epileptogenic networks are largely supported by findings of simultaneous spikes in distant areas.

More than 50% of the epileptic spikes in the mesial temporal region are synchronously expressed in the neocortical temporal regions (30–32). In addition, mesial temporal structures have shown an increased tendency to synchronize during interictal activity (31, 33). Resting-state studies have shown that there are established connections within the limbic system, with interactions between the amygdala, anterior hippocampus, entorhinal cortex, and the posterior hippocampus (34). Some suggest that these patterns could parallel findings in the kindling models described in rats as part of the pathophysiology of TLE. BOLD fMRI connectivity studies with SEEG registration have found that there is a trend for fMRI with functional connectivity reduction in the EZ and the interictal zones, potentially secondary to an alteration in the neurovascular coupling (30).

## Ictal SEEG in Temporal Lobe Epilepsy

Ictal SEEG activity has been studied widely, especially regarding fast activity seen as low voltage fast activity (LVFA) described by Bancaud in 1965 (6). Visually, LVFA is readily visible when interpreting SEEG, as it disrupts the background. The unique properties of the limbic electrophysiological properties need to be kept in mind to better determine abnormal onset activities (30). Specifically LVFA usually indicates a lesion that is highly epileptogenic. Slow or sharp waves are seen intermixed in the LVFA, often in the ascending or descending portions of the wave. In the case of hippocampal onset seizures, repetitive spikes or sharp waves may summate/build up as part of the preictal phase, especially in these regions, and are associated with focal cortical dysplasia (FCD) (35, 36).

“Lead-in” structures in the epileptogenic zone play an important role in establishing seizure networks. The time lag between the “leader” structure and propagated structures helps to understand the epileptic networks (37). A study by the Yale group found that patients with drug-resistant TLE, especially involving the anterior temporal region, with seizure propagation in <10 s had a higher rate of surgical failure (hazard ratio (HR), 5.99; 95% CI, 1.7–21.1; *P* < 0.01) (38). Distance between the electrodes may also affect the latency of propagation and further studies are needed to validate this finding.

In mesial temporal lobe epilepsy (MTLE), low frequency and high amplitude periodic spikes with a hypersynchronous onset have been associated with neuronal loss and gliosis in this area, especially in the hippocampal formation (39, 40). In addition, hippocampal spikes tend to spread to the entorhinal cortex and amygdala, and posterior cingulate area (41). Characteristically, in MTLE seizures, symptoms occur late after onset within the mesial limbic regions, manifested later as the propagation of the seizure spreads out of the EZ (2, 19).

Subclinical or electrographic seizures may be observed as rhythmic or fast activity similar to a focal discharge that may appear in one contact or regionally, lasting more than 10 s. At times, this activity is similar to the initial portion of a seizure that has clinical manifestations when spreading to other structures (6). Importantly, subclinical seizures have been deemed a high localizing finding and if resected, positive post-surgical outcomes are reported (42).

Postictal findings are relevant, especially in the early stage, where focal attenuation/ suppression of background may link to the epileptogenic zone. The longer the suppression/attenuation lasts, the more pathologic the EZ might be (26, 30, 43).

## THE Relevance of Fast Activity: Ripples and High-Frequency Oscillations

Animal studies suggest that the common epileptogenic patterns in MTLE are periodic spikes and LVFA (Figure 3D). These may differ in high-frequency oscillations (HFO) characteristics. High-frequency waves have been associated with highly epileptogenic networks. Gamma oscillations are defined as those within 30–80 Hz (seen in the hippocampus) and are a physiological representation of the balance of excitation and inhibition in local neurons. Those classified as HFOs are 125–500 Hz, and very HFOs (vHFOs) (250–500 Hz) (44). Some conflicting evidence exists on the previous findings, as these may also be called ripples and fast ripples; physiological findings. Another study group advocates that fast ripples increase during the onset of seizures as periodic spikes and ripples predominate in those seizures beginning with LVFA (45).

## Types of Seizure Patterns in SEEG: THE Old and The New

Perucca et al. published findings on seizure onset patterns in patients with SEEG. These patterns are as follows: 1. LVFA is defined as rhythmic low voltage (<10 μV) activity above 13 Hz; 2. Low frequency, high-amplitude periodic spikes, at.5–2 Hz; 3. Sharp activity ≤ 13 Hz, low to medium-voltage sharply-contoured rhythmic activity in the alpha-theta range; 4. Spike and waves, medium to a high voltage ranging in the frequency of 2–4 Hz; 5. A burst of high-amplitude polyspikes, characterized by a single brief burst of repetitive high-voltage spikes; 6. Burst suppression is seen as brief bursts of medium- to high-voltage repetitive spikes alternating with brief periods of voltage attenuation; 7. Delta brush is seen as rhythmic delta waves at 1–2 Hz, with superimposed brief bursts of 20–30 Hz activity overriding each delta wave. Low frequency high-amplitude periodic spikes (type 2 pattern) were specific to mesial temporal atrophy or sclerosis and were seen only in the mesial temporal lobe. When analyzing the temporal region, LVFA (type 1 pattern, seen most repeatedly), low frequency high-amplitude periodic spikes (type 2 pattern), and sharp activity (type 3 pattern) were seen. These were also three of the four patterns identified in temporal atrophy or sclerosis, although there were no significant differences in the frequency of the three patterns between seizures that arose from pathological tissues vs. healthy mesial temporal structures. Other anomalies associated with these patterns in the study were FCD, periventricular nodular heterotopia, tuberous sclerosis complex, and cortical atrophy (41). These results were recently replicated by a Chinese group, finding LVFA was most common, present in more than 40% of cases with TLE (46).

In another study, cases were divided into an MTS group and a non-MTS group based on imaging. Seizure onset patterns were analyzed and classified to determine the correlation between surgical outcome and clinical subtypes. Five seizure onset patterns were determined for MTLE, and 2 were correlated with MRI findings. Multiple seizure onset patterns did not predispose to poor outcomes in this subgroup, but multifocal seizure onsets especially when outside the resected area in the temporal region had poor outcomes (13). In a separate analysis by Lee et al. (47), neocortical patterns of epilepsy in the pediatric population were explored. Regional onset was most seen in the gamma range and temporal, or focal onset was seen in beta frequency ranges or slower. LVFA was once again the most common form of seizure onset activity in 57% and was most frequently seen in developmental pathological findings. In comparison, rhythmic sinusoidal waves at onset were found in only mature cases. In addition, LVFA and rhythmic sinusoidal wave onset patterns were associated with favorable and slow onset suggesting poor outcomes in the subgroup of developmental pathology (47).

One of the indications of SEEG is establishing the laterality of the EZ when scalp EEG and non-invasive information are discordant. SEEG recordings involving the hippocampal region correctly identify and lateralize temporal lobe seizures far more than limited subdural electrodes (42). A recent study using SEEG revealed that only 4/14 (29%) patients indeed had unilateral TLE. In addition, 2 distinct SEEG patterns of seizures were described: a temporo-mesial origin of seizures, and multiple onset zones in the mesial and lateral temporal cortex or from the extra-temporal cortex. Patients in the temporo-mesial pattern underwent surgery and had a favorable outcome. The laterality of the seizures was not the only element considered when deciding which side to intervene, but the non-invasive information played a crucial role as well (48).

## Presurgical Cortical Stimulation

Cortical stimulation (CS) is one of the mainstays of the study of the EZ, the elements surrounding it, and the capability of surrounding tissue to cause seizures or be pro-epileptogenic. Presurgical stimulation is not done routinely in all comprehensive centers, but it is certainly encouraged in most pioneering centers worldwide (2, 7, 49, 50). CS allows comparing induced seizures with spontaneous seizures to precisely localize the EZ and its boundaries. Any component of the EZ is in theory able to synchronize the whole network, and stimulation along these areas might trigger a seizure. Identification of LVFA during stimulation may reveal networks that were unclear spontaneously. In addition, seizure architecture may be different when stimulating different elements of the EZ and may trigger different seizure paths in the same network. This is especially true in regional neocortical temporal epilepsies that involve the occipital/temporal or occipital/temporal/perisylvian regions. The order of activation of the components of the EZ is important to establish propagation patterns.

In addition, after discharges (AD) are pivotal in CS. When activating AD, the team may obtain information about epileptogenic areas of interest and correctly discern them from non-epileptogenic tissue. Specifically, in mesial temporal regions, single or trains of pulses are delivered to stimulate the hippocampus at low amperage, due to its low threshold for stimulation (51). Pulses (single.3–0.5 ms or trains for up to 5 s) with an intensity preferably of 0.5–5 mA are delivered. The same intensity is applied to known affected tissue with pathology, like dysplasia (5, 19, 52). A key fact in CS is not to stimulate repeatedly in the same contact line, as the epileptiform discharge may be “fatigued”. AD morphology has been studied, but not found to be related to a specific subtype of epilepsy, or EZ. Blume et al. described 5 different morphology patterns in SDEEG: sequential spikes with pauses, spikes-waves at 1–3 Hz, polyspike bursts, rhythmic waves and spikes, and rhythmic waves (53). They were described in mostly temporal regions using subdural recordings. However, in this SDEEG study, the authors found that when AD involved more than the stimulus site it might lateralize incorrectly and be misleading. AD that is triggered outside the known EZ are important findings and signifies non-synchronization of that specific area.

In the case of the infra-Sylvian epilepsies (temporal and occipital), the mode of organization is similar according to a study by Chauvel. In this subgroup, the elements of the “typical” seizure may be obtained and the peculiar hyperexcitability and hyperconnectivity of the limbic system make this area complex for interpretation. When compared to its supra-Sylvian counterpart that can trigger seizures without the presence of AD, as an all or- non-phenomenon, the limbic system involved in epilepsy is complex (54). A French group found that when stimulating the insular cortex, two networks were seen: a visceral network that extended to the temporo-mesial structures and a somesthetic network that reached the opercular cortex (55).

Contradicting evidence exists on the advantages of presurgical stimulation. The estimation of the concordance between stimulation-induced and spontaneous seizures was 90% for temporal regions in a recent study (13). A separate group found that afterdischarges could not be used as a standardized tool to localize EZ (2). False negatives were abundant and difficult to interpret. Due to this finding, CS is not a standard in some centers and direct stimulation on the surgery day is a common practice (2). Recently, in a joint study by the MNI and Grenoble groups, 103 patients were studied, and seizures induced by CS identified the EZ and its primary generator as reliably as spontaneous seizures. They suggested using CS in a more time-efficient manner could reduce hospital stays and potential morbidity (50).

Cortical stimulation is also done to establish the limits of the functional or eloquent brain areas. The main targets of interest are mapping of language, especially with the close relationship with opercular structures. The anterior and posterior language areas belonging to the dorsal language stream share characteristics with primary cortices, basal temporal regions, and high associative ventral temporal regions. The need to understand the language as a network and not a single area is important during CS (54). In cases of lesional epilepsy, the stimulation of zones farther away from “conventional” language areas is seen and can certainly influence surgical decisions. In fact, language mapping that finds the involvement of the basal temporal language area involved in this region must be taken into consideration. When this area is resected, a decline in naming is seen and persists across time (56). Figure 3 depicts a case that exemplifies the SEEG vision of TLE.

## Pitfalls and Complications of SEEG

While we have established the advantages of SEEG recordings, their use must be reserved for suitable cases. The caveat of SEEG implantation is always avoiding “fishing expeditions”, a common term used to depict extensive implantation of both hemispheres, likely due to poor non-invasive information or contradicting information. Although some cases are thoroughly complicated, especially those with late-onset, mesial frontal involvement, or temporal plus characteristics (5). A fishing expedition further complicates SEEG interpretation, as the propagation pattern is difficult to analyze, and finally, the latencies between areas may not be adequately weighed. Implantations that exceed 15 depth electrodes have a calculated risk of complication of 0.18%. per electrode (57). As was mentioned earlier, latencies are very important to establishing a surgical prognosis (38). While the planned electrodes are placed on the selected cortical areas that are suspected to originate from the epileptogenic zone, their area of coverage is so precise that the main area of seizure onset may be missed (5).

The capability of brain mapping is restricted in SEEG when compared to the cortical superficial mapping of SDEEG. SDEEG has a convenient view of the superficial neocortex, but the feasibility of the stimulation for functionality in SDEEG has not been formally studied when comparing it to SEEG. SEEG allows a three-dimensional view of the epileptic network, a task that has yet not been obtained by just SDEEG. Finally, when the goals of treatment are palliative SEEG is greatly discouraged. Selection of patients involves not only seizure patterns and propagation. Patients that are cognitively or behaviorally impaired may not be adequate candidates for SEEG, as they may explant or cause self-lesions (58).

Complications of SEEG implantation are low. They mainly occur in the implantation and explanation process. Most comprehensive epilepsy centers report no complications with SEEG implantations (5, 15, 20, 24). In a systematic review done by the Cleveland team, SEEG was considered a safe procedure; the most common complications were hemorrhage (1%, 95% CI 0.6–1.4%) and infection (0.8%, 95% CI 0.3–1.2%) (59). While SEEG is most frequently used in comprehensive epilepsy centers and is ultimately replacing SDEEG, a recent study showed thatpatients that underwent SDEEG were more likely to undergo surgery, but not obtain seizure freedom and had a higher odd of complications related to the procedure (OR = 2.24, 95% CI 1.34, 3.74; unadjusted: 9.6% after SDE vs. 3.3% after SEEG). Seizure freedom was higher in the SEEG group as well (60). Although SEEG is safe for the pediatric group of patients, it is not a common practice to undergo SEEG implantation before ages 2–3 years of age (14, 21).

## SEEG in NON-lesional vs. Lesional Temporal Lobe Epilepsy:

Non-lesional TLE is evolving as a unique entity of TLE. It represents seizures originating from the temporal lobe based on electroclinical findings and semiology, in the absence of an epileptogenic lesion on the MRI (MRI Negative). On the contrary, lesional TLE (MRI–positive) is defined as the presence of hippocampal sclerosis (HS) as the most common pathologic (65–70%) finding in (MTS), or other lesions confined to the temporal lobe (61).

Distinguishing seizures from mesial, neocortical TLE, or deeper anatomical focus can be challenging. They can be clinically “silent” areas and can produce seizures with semiology and electrophysiological findings indistinguishable from MTS, including the temporal pole (62), orbitofrontal cortex, insula, posterior cingulate gyrus (63), and temporo-parieto-occipital area (64). The distinction is essential because resection of only the mesial temporal structures in these patients will not control the epileptic seizures. Physicians must be cautious to consider resection for TLE when images are unremarkable. In general, TLE with “normal” MRI requires more investigations (Phase I and II) before a surgery decision can be made. Accurately identifying the absence of a possible epileptogenic structural lesion is crucial. This can change both the indications of SEEG and surgery outcomes. Patients must be investigated carefully with a high-resolution epilepsy protocol MRI with both T1 and T2-weighted images. Challenge in confirming a diagnosis of MRI-negative TLE is that accurate classification relies upon the quality of the MRI and the radiologist's expertise in interpreting those images (Table 2).

**Table 2:** Indications of SEEG in lesional and non-lesional temporal lobe epilepsy.

**Non-lesional Temporal Lobe Epilepsy**	**Lesional Temporal Lobe Epilepsy**	**Both Lesional and Non-lesional Epilepsy**	
Dominant Temporal Lobe Epilepsy and normal pre-surgical memory: • Explore sparing the hippocampus if not involved in seizure generation to reduce the risk of post-surgical verbal memory decline (65)	Multiple lesions Uncertainty of involvement of bilateral temporal, “*pseudo*-temporal”, or temporal-plus epilepsies (in discordant electro-clinical manifestations). “Dual pathology”: hypothesis suggests one epileptogenic zone and not multifocal epileptogenic zones, (66) Central hypothesis of the EZ does not coincide with lesion identified on MRI (67)	**Clinical**: • Aura of unusual presentation of mesial temporal semiology, suggesting lateral or extra-temporal onset. **Electroencephalogram**: • Interictal scalp electroencephalogram with bilateral temporal spikes • Interictal scalp electroencephalogram with posterior temporal spikes or extra-temporal/neocortical spikes. • Ictal scalp electroencephalogram with unclear seizure onset, extra temporal onset, and/or originating in one temporal region and propagating quickly to contralateral temporal region.	**Neuropsychological evaluation:** • Neuropsychology testing suggestive of functional deficit in bilateral, extra temporal or contralateral temporal regions. **Imaging (additional):** • Negative/discordant functional imaging (Positron Emission Tomography and/or Single- Photon Emission Computed Tomography).

Interpreting scalp EEG by a seasoned electroencephalography expert is mandatory in every case. For instance, ictal frequency of 2–5 Hz irregular rhythm with widespread temporal distribution suggests either neocortical or a deeper epileptogenic origin of seizures. For these cases, SEEG may be especially useful. In cases of suspected extratemporal lobe epilepsy, IEDs can be seen over precentral, bilateral, anterior medial temporal (identical to Mesial TLE), or without any IED (68). Planning for further SEEG investigation, especially when non-concordance hypotheses arise, depending on their overall clinical, imaging, and EEG findings may be warranted (69). In fact, in a cohort of 177 patients with SEEG, 29 had non-lesional MTLE. They proposed a standard bilateral limbic coverage when implanting all non-lesional cases (70). Most centers apply individualized implantation depending on a specific seizure propagation pattern and clinical characteristics and not necessarily bilateral limbic coverage in all cases.

Furthermore, nuclear medicine imaging is complementary in localizing and/or lateralizing the EZ, aiding in SEEG implantation planning and covering the most possible anatomical areas with the highest epileptogenicity. These modalities can be used as independent predictors of seizure freedom outcomes after surgery (71). Positron emission tomography (PET) is an interictal study that helps in SEEG pre-implantation mapping by identifying the hypometabolic areas which may highlight focal regions of cortical dysfunction. PET frequently shows an area of hypometabolism extending beyond the EZ. Thus, physicians cannot use it solely to delineate surgical margins of resection. Nevertheless, it remains of value for lateralization and general localization of epilepsy in all cases mainly negative MRI for the planning of SEEG implantation (72).

Additionally, Ictal-interictal SPECT (Single-Photon Emission Computed Tomography) provides a high yield as a non-invasive option for non-lesional cases. It provides an indirect measurement of the increase in cerebral blood flow (CBF) during ictal epileptic activity (73). Studies determined that when comparing the utility of both SPECT and PET in identifying the EZ, SPECT is superior to PET in identifying the epileptogenic zone in both lesional and non-lesional TLE. In MRI-negative cases, SPECT showed 64% sensitivity, while PET only 36%. Contrasting with MRI-positive TLE cases, the sensitivity of SPECT was 87.5% and in PET 62.5% (74). PET and SPECT provide no statistical additional localizing value if electro-clinical and MRI findings are concordant (70).

The role of neuropsychological evaluation in the localization of the seizure and the pre-implantation mapping remains controversial. Studies involving TLE non-lesional cases emphasized that normal memory does not preclude seizure onset in any focus within the temporal lobe, specifically the mesial TLE. Therefore, they are mainly used to predict verbal memory outcomes after surgery is planned (75).

Non-lesional TLE patients can have up to 56% seizure freedom when adequately selected (70). Some centers favor implanting full bilateral temporal SEEG coverage in those cases even if the pre-implantation hypothesis is highly suggestive of unilateral focus (48, 76). In one study, up to 14% were ultimately determined to have independent bitemporal seizures despite a unilateral non-invasive evaluation (70). Other centers have found favorable outcomes with bilateral temporal (biTLE) SEEG coverage in TLE with known unilateral hippocampal sclerosis when the possibility of a true biTLE was high (77).

## Outcomes of SEEG Implantation in Temporal Lobe Epilepsy

Resective surgeries and SEEG implantation techniques are evolving rapidly (19, 78). In general, patients that do not undergo resection after SEEG implantation and recording are up to 40%. Particularly in non-lesional TLE, this group had lower outcomes after surgery with or without SEEG implantation. For example, seizure freedom rates in bilateral—SEEG implantation patients were (32%) compared to those unplanned for SEEG and to unilateral SEEG implantations (43%)(79). Some studies have found possible predictors of epilepsy outcomes after SEEG implantation. Negative predictors of seizure freedom after SEEG are more than one seizure focus, seizure-free periods in their medical history, and a non-localizing ictal scalp EEG. On the other hand, a positive prediction of seizure freedom was observed with identified MRI lesion or PET hypometabolism in concordant with a strong and testable anatomo-functional hypothesis (80, 81). Another outcome series by the Cleveland group aimed to determine the rates and predictors of seizure freedom after resection among bilateral SEEG implanted patients. Observed positive predictors were single seizure type, short epilepsy duration (<10 years), absence of bilateral independent ictal seizure onset, and presence of dominant IEDs, which were among the most significant (76).

For patients that have been treated with failed resection of temporal regions, SEEG can also establish EZ characteristics for further treatment strategies and decision-making. In a study of post-surgical TLE patients in Germany from 2013 to 2017, the investigation of 21 patients that required SEEG led to a change in the initial surgical plan in more than 60% of patients, with resection area ranging in over 81% and an outcome of Engel I in 75% of patients (82). In fact, in a recent retrospective study of 85 patients that underwent anterior temporal lobectomy with bilateral TLE or poor lateralizing data, the SEEG study yielded equivalent outcomes in unilateral TLE (83).

Additionally, one of the reasons for surgery failures in TLE raised attention to the speed of propagation of seizure activity recorded intracranially in both groups of unilateral and bilateral TLE hypotheses. Lieb et al. (84) reported that an interhemispheric propagation time <5 s was negatively correlated with seizure outcomes. A mean interhemispheric propagation time of 39 s was associated with a favorable outcome. A recent study showed that rapid seizure spread in <10 s was associated with recurrence of seizures, despite 61% of those patients being with lesional TLE (38). Overall, reported outcomes could be controversial given the fact variations between the centers in patient selection criteria, implantation data processing, and available diagnostic techniques.

Stereo-encephalography implantation is useful to establish EZ that is extratemporal in origin. In a retrospective case series using SEEG patients implanted to discern TLE vs. Extra-TLE and finally found to have extra-TLE were described. In adequately established extra-TLE, surgical results were favorable in all cases at follow-up (85).

## SEEG For Treatment: Intraoperative Guidance, Laser Ablation, and Thermocoagulation

Laser ablation has become a common treatment mode combined with SEEG guidance. Lesions related to TSC, MTS, FCD, hamartomas, and strokes have all been successfully treated with laser ablation. Laser ablation offers a small opening required to insert the probe, and high precision for the direction of the laser probe, with <5 min required to perform the procedure. This procedure targets the amygdala, hippocampus, subiculum, and part of the entorhinal cortex. (Standard ATL includes the previously mentioned structures, but also the temporal pole, the entorhinal cortex in the fusiform and parahippocampus, and the lateral temporal region. This extension on resection conveys adequate seizure freedom results.) (86). In a study involving 10 patients with 15 distinct areas of epileptogenicity and a robot assistant, all patients were discharged the next day after the procedure, 5 patients had a seizure outcome of Engel I without major complications (26). Laser interstitial thermal treatment (LiTT) has become of interest as part of the treatment in comprehensive epilepsy centers. A recent systematic review and meta-analysis compared outcomes of MR-guided LiTT (MRgLiTT) and SEEG-guided radiofrequency (RF) thermocoagulation (SEEG-RFTC). Outcomes were efficacious for MRgLiTT in hypothalamic hamartoma and failed anterior temporal lobectomy. However, patients with TLE and MTS did not achieve better seizure outcomes than non-MTS patients. Current evidence with LiTT is limited, with case series and ongoing research interest, with the use of robotic implantation to decrease surgical morbidity (86). LiTT is not available in all comprehensive epilepsy centers and is not a standard practice.

Thermocoagulation has long been used in European comprehensive epilepsy centers as a treatment for lesional temporal epilepsy with promising results. The “classic” mainstay of resective surgery involving the mesial temporal region, especially with MTS, is anterior temporal lobectomy (ATL). However, ATL presents with occasional side effects concerning visual field defects and memory impairment. SEEG- guided radiofrequency thermocoagulation (RF-TC) provides an alternative treatment with inferior seizure freedom when compared to ATL, but superior results with respect to function. A Chinese study also found that RF-TC for MTLE with MTS was well-tolerated in 22 cases and most had a 90% decrease in seizure frequency in 12 months (87). In contrast, a French group studied 21 patients that underwent SEEG-guided RF-TC and compared them to 49 patients that underwent ATL. No patients on the RF-TC were seizure-free at 12 months; 37 (75.5%) were seizure-free in the ATL group. They recommended this procedure be reserved for difficult to treat dominant and/or non-resectable MTS cases (88). Trials are currently ongoing to establish differences between these subgroups.

**Figure 4:**
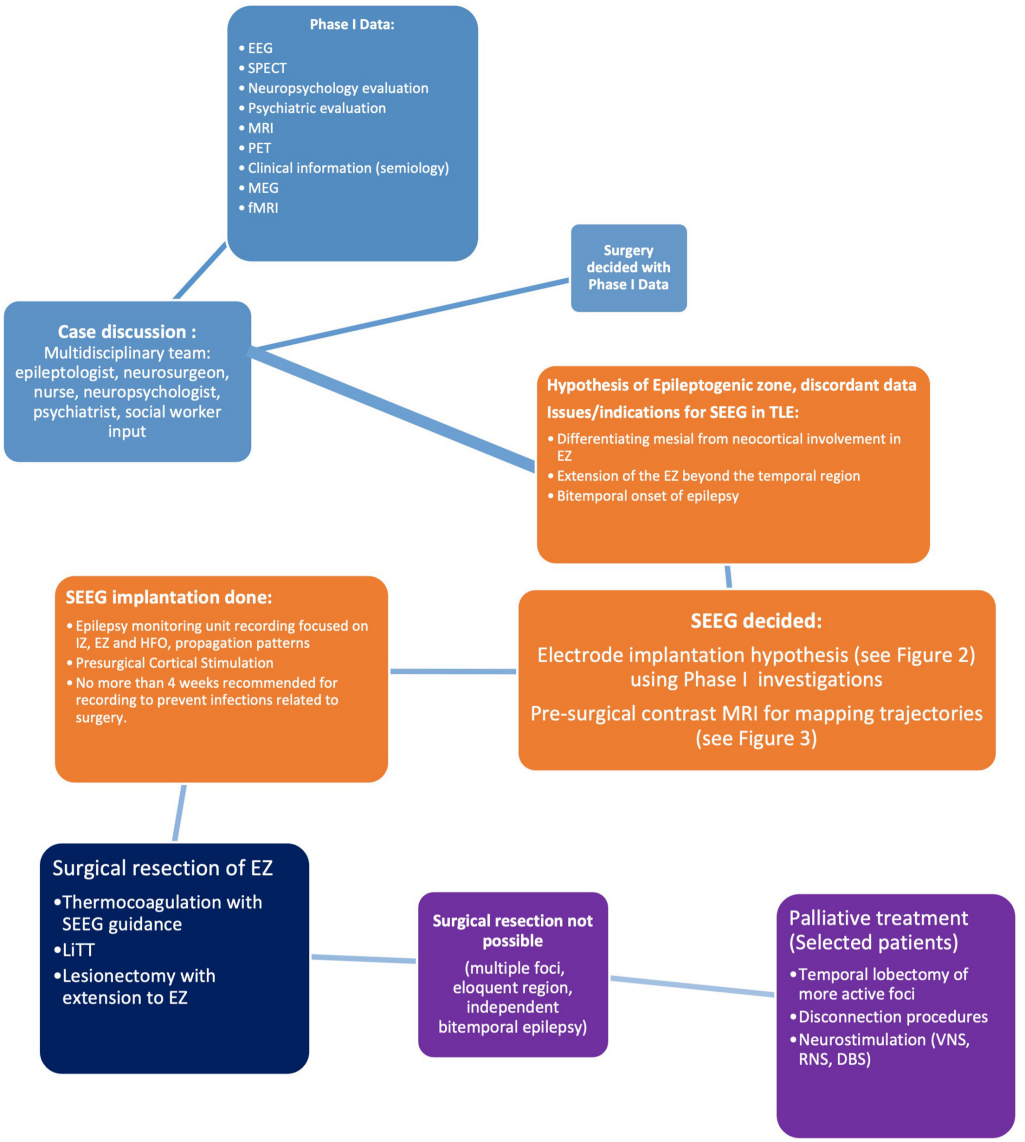
Proposed algorithm for temporal lobe epilepsy investigations using SEEG.

In SEEG-RFTC cases, patients with periventricular nodular heterotopias obtained the highest rate of seizure reduction. The rate of complications of both procedures was low (<5%) (89). A recent study suggested RFTC could be used as a segue to secondary interventions and for heterotopias. In cases where the language dominant side is involved, they proposed a minimally invasive treatment with multiple hippocampal transections using RF-TC (90). This is not yet a common practice in all Comprehensive Epilepsy Centers (Figure 4).

## Artificial Intelligence Applied to SEEG and Future Directions

One of the most rapidly increasing sciences is artificial intelligence (AI). SEEG is currently used as part of the algorithms to predict seizures. One study used a stacked one-dimensional convolutional neural network (1D-CNN) model combined with a random selection and data augmentation (RS-DA) strategy. They used this method to study scalp and intracranial EEG. For the scalp EEG detection, an 88.14% sensitivity, 99.62% specificity, and 99.54% accuracy, and for SEEG, 90.09% sensitivity, 99.81% specificity, and 99.73% accuracy were obtained. They concluded that the prediction model was accurate (91). A miniature robotic model system has also been encouraged as part of the SEEG process of electrode implantation, with a more precise implantation system and shorter timeframes (92). Deep learning models have been developed to identify epileptogenic signals from the epileptogenic areas of the brain. They use raw time-series signals to build a one-dimensional convolutional neural network (1D-CNN) to achieve end-to-end deep feature extraction and signal detection. A sensitivity of 97.78%, an accuracy of 97.60%, and a specificity of 97.42% were found in the Bern–Barcelona database. Deep learning may pivot new standardized studies for automated SEEG seizure detection systems (93).

The growing field of epilepsy is flourishing especially with the standardized SEEG used in Comprehensive Epilepsy Centers. One study used BOLD signal and resting-state fMRI to reflect brain pathological regions and epileptiform discharges. Functional connectivity (Fc) using fMRI was cross-related with SEEG in 5 patients with DRE and TLE. Fc signal was more prominent in regions affected by epileptiform abnormalities. Significant negative correlations were found between the FC of SEEG and BOLD signal when considering all pairs of signals (theta, alpha, beta, and broadband), suggesting differential effects of epileptic phenomena secondary to pathological plasticity in TLE (29). HFOs are now being studied as a part of the seizure prediction tools. Using machine learning algorithms, detection systems used SEEG signals and HFOs to recognize patterns that preceded seizures by up to 30 min. Not only can HFOs help predict seizures, but a study suggests that they can even predict acute development of a first seizure and chronicity of TLE (44).

## Discussion

Temporal lobe epilepsy is the most frequent and most studied of the DRE epilepsies. Its treatment with surgical resection has shown promise for seizure freedom. Since its initial description in 1957 by Bancaud and Talairach, a lot has changed. From the original notion of seizure onset zone and its interaction with the irritative and lesional zone to the theory of epilepsy as a network, SEEG has created an evolution in the way we see epilepsy.

Indicated in “difficult to localize” epilepsies, SEEG permits both the clinician and the surgeon to understand the interaction of pathologic epileptogenic networks and physiologic pathways. Surgical planning is precise and establishes boundaries of resection with optimal responses. With the description of the atlas by the Montreal Neurological Institute, we can now establish the baseline for SEEG neuronal activity (16). Morphology of SEEG interictal and ictal activity has long been studied and will continue to grow as machine learning and AI influence and optimizes seizure detection. The exciting studies comparing imaging and co-registering SEEG for the treatment of TLE in a tailored manner promise great advances in the years to come. Perhaps in the future, SEEG will be optimized by early CS, machine learning for seizure detection, and finally tailored, non-invasive treatment with excellent results.

## Author Contributions

EP-A researched, edited, and drafted the manuscript, and adapted figures and images. NA researched, edited, and drafted the manuscript. DB-H provided images and resected brain tissue pictures. SM supervised, revised, and edited the manuscript. All authors contributed to the article and approved the submitted version.

## Funding

This study was funded by the Department of Clinical Neurological Sciences in London Health Sciences Centre, London, Canada.

## Conflict of Interest

SM has received honorarium for speaking engagements for UCB Canada Inc., Eisai Co. Ltd. (Canada), and Sunovion Pharmaceuticals Canada Inc. He has been member of the Epilepsy National Advisory Board for UCB Canada Inc., Eisai Co. Ltd. (Canada) and Sunovion Pharmaceuticals Canada Inc. He has been involved in multiple multi-center clinical trials unrelated to the subjects addressed in this paper. The remaining authors declare that the research was conducted in the absence of any commercial or financial relationships that could be construed as a potential conflict of interest.

## Publisher's Note

All claims expressed in this article are solely those of the authors and do not necessarily represent those of their affiliated organizations, or those of the publisher, the editors and the reviewers. Any product that may be evaluated in this article, or claim that may be made by its manufacturer, is not guaranteed or endorsed by the publisher.
